# Inhibiting biofilm growth on ammonium salt-functionalized or fluorinated voice prostheses silicone

**DOI:** 10.1007/s00253-026-13904-z

**Published:** 2026-06-30

**Authors:** Sławomir Okła, Szczepan Kaliniak, Jakub Spałek, Katarzyna Piotrowska, Dawid Łysik, Piotr Deptuła, Konrad Żochowski, Michał Dutkiewicz, Joanna Mystkowska, Urszula Wnorowska, Robert Bucki, Bonita Durnaś, Monika Madej, Hieronim Maciejewski, Stanisław Góźdź

**Affiliations:** 1 Holy Cross Cancer Center, Artwińskiego 3, 25-734 Kielce, Poland; 2 Institute of Medical Science, Collegium Medicum, Jan Kochanowski University of Kielce, IX Wieków Kielce 19A, 25-317 Kielce, Poland; 3 Faculty of Mechatronics and Mechanical Engineering, Kielce University of Technology, Tysiąclecia Państwa Polskiego 713904, 25-314 Kielce, Poland; 4 Institute of Biomedical Engineering, Bialystok University of Technology, Białystok, Poland; 5 Independent Laboratory of Nanomedicine, Medical University of Białystok, 15-222 Białystok, Poland; 6 Department of Medical Microbiology and Nanobiomedical Engineering, Medical University of Białystok, 2 C A. Mickiewicz Str., 15-222 Białystok, Poland; 7 Poznań Science and Technology Park, Adam Mickiewicz University Foundation, Rubież 46, 61-612 Poznań, Poland; 8 Faculty of Chemistry, Adam Mickiewicz University, Uniwersytetu Poznańskiego 8, 61-614 Poznań, Poland

**Keywords:** Voice prosthesis, Silicone, Functionalized silicone, Total laryngectomy, Biofilm, Complications

## Abstract

Voice prostheses (VPs) are largely limited by their relatively short functional lifespans, mainly due to colonization by heterogeneous fungal-bacterial biofilms. This biofilm-mediated degradation is a major contributor to VP failure and is associated with clinically significant complications such as impaired phonation, leakage through the tracheoesophageal fistula, and an increased risk of pneumonia. We performed a series of in vitro experiments to assess the antimicrobial and physicochemical properties of VPs constructed from quaternary ammonium salt–functionalized or fluorinated silicone. Biofilm development on each prosthesis was quantified by measuring crystal violet–stained biomass, and surface coverage was visualized using scanning electron microscopy. Furthermore, biofilm morphology and material adhesion characteristics were examined by atomic force microscopy. To evaluate cytocompatibility, fibroblasts were exposed to extracts obtained after 24 h incubation of each material in culture medium. This was followed by analysis of cell area, circularity index, haemolytic activity and cytokine secretion. Mechanical testing was performed to determine the physiochemical performance of the tested silicone materials. Crystal violet staining demonstrated substantial biofilm accumulation on VPs retrieved from patients after three months of use. In contrast, the modified silicone materials exhibited markedly reduced biofilm coverage. All tested materials supported normal fibroblast proliferation and showed minimal hemolytic activity, although they differed in their capacity to induce cytokine expression. Incorporation of ammonium salt-functionalised or fluorinated silicone in VP design may substantially reduce surface biofilm formation. Thereby, improving device longevity and decreasing the risk of use-related complications.

**Table:** 

**Key points** • *Fluorinated and quaternary ammonium salt–modified silicones exhibit significant biofilm resistance.* • *The modified materials demonstrate established biocompatibility with human cells.* • *These findings suggest the potential for developing new, extended-lifespan voice prostheses.*

**Supplementary Information:**

The online version contains supplementary material available at https://doi.org/10.1007/s00253-026-13904-z.

## Introduction

Total laryngectomy (TL) results in the complete loss of phonation abilities. The current standard for post-TL voice rehabilitation is the surgical creation of a tracheoesophageal fistula (TEF) with concurrent implantation of a voice prosthesis (VP) (Souza et al. 2020). VPs, typically manufactured from silicone-base polymers, function as a one-way valve that permit airflow from the trachea into the oesophagus to enable phonation (Spałek et al. 2022). Despite the high effectiveness of this method, the use of VPs, and silicone medical devices in general, remains associated with several complications. Silicone is a material widely used in biomedical applications due to its favorable physicochemical properties, including low thermal conductivity and chemical reactivity, high biocompatibility, and low toxicity. It has been adopted across numerous medical fields, from short-term devices such as urinary and central venous catheters, to long-term implants including artificial joints. Silicone materials exhibit excellent thermal stability across a broad temperature range (− 100 to 250 °C), hydrophobicity, and resistance to creasing, wrinkling, oxygen, ozone, and ultraviolet light. Although they adhere poorly to many substrates, they show strong adhesion to others, such as glass (Moretto, Schulze et al. n.d., Laskovski 2011; Song et al. 2018).

The National Institutes of Health estimates that biofilms are involved in more than 80% of all microbial infections in the United States (Nobile and Johnson 2015). A major limitation of voice prostheses (VPs), as well as other silicone-based medical devices, is their susceptibility to fungal and bacterial colonization, which leads to biofilm formation and ultimately limits device longevity (Douglas 2003, Kojic and Darouiche 2004, Sayed, Datta et al. 2012, Galli et al. 2018). The average functional lifespan of a VP ranges between approximately 2 and 10 months (Op de Coul et al. 2000; Mäkitie et al. 2003; Lam et al. 2005; Bozec et al. 2010; Hancock et al. 2013; Kress et al. 2014; Thylur et al. 2016; Lewin et al. 2017; Serra et al. 2017; Mayo-Yáñez et al. 2018; Petersen et al. 2019; Martins de Sousa et al. 2022). Within the biofilm structure, *C. albicans* cells express surface adhesion molecules such as Eap1 and Als3, which function as specific receptors for bacterial attachment, including for strains of *Streptococci* spp. and *Staphylococcus aureus*. This cooperative adhesion contributes to the predominance of mixed bacterio-fungal biofilms on silicone medical devices. Colonization and subsequent biofilm maturation, by *Candida* spp., including *C. albicans*, *Pichia kudriavzevii* (formerly *Candida krusei*), *C. glabrata, C. tropicalis*, play a critical role in the degradation and failure of VPs (Nobbs et al. 2010; Peters et al. 2012; Spałek et al. 2020). *Candida* spp. form complex three-dimensional networks that disrupt proper valve function and compromise prosthetic device performance (Ell 1996; Bertl et al. 2012). Hyphal adhesion of *Candida* spp. is essential for initiating biofilm development, and penetration of yeast filaments is facilitated by enzymatic degradation of silicone, which may release nutrients that further support fungal growth (Finkel et al. 2012; Lagree et al. 2018). Both genetic characteristics of *C. albicans* and external factors such as surface topography and host immune responses strongly influence fungal adherence (Ell 1996; Van Weissenbruch et al. 1997). Additional factors such as reactive oxygen species and extracellular enzymes produced by monocytes and neutrophils contribute to silicone deterioration (Babior et al. 2002). Therefore, biofilm growth is the primary driver of deformation, structural damage, and functional failure of VPs. Compromise of VP integrity is associated with various complications, some of which may be life-threatening. Central and lateral leakage of the tracheoesophageal fistula is the most common issue and can result in aspiration pneumonia. Other previously reported complications include leakage of oesophageal contents, valve malfunction, granulation tissue formation, cervical cellulitis, necrosis of the tracheoesophageal puncture, tracheostomal stenosis, and swallowing difficulties (Van Den Hoogen et al. 1996; Laccourreye et al. 1997; Lewin et al. 2017; Spałek et al. 2021). Various interventions have been explored to extend VP lifespan by limiting biofilm formation, such as nystatin therapy, antimicrobial agents, oral rinses, and mechanical cleaning with specialized brushes. None of these methods have demonstrated fully satisfactory long term efficacy (Ameye et al. 2005; Messing et al. 2015). In otorhinolaryngology silicone polymers are also used in other biocompatible materials, including cochlear implants and tympanostomy tubes. These are also susceptible to biofilm-associate complications such as the formation of *P. aeruginosa* biofilm, development of methicillin-resistant *S. aureus* otorrhea following tympanostomy, tube occlusion, and patient discomfort (Jang et al. 2009, 2010; Spałek et al. 2022).

Fungal biofilms on medical devices act as persistent reservoirs for pathogenic microorganisms and can lead to both localized and systemic infections (Carolus, Van Dyck et al. 2019). Device-associate biofilms are a major cause of severe clinical complications; for example, approximately 90% of catheter-related infections require hospitalization, and up to 70% of *Candida* spp. bloodstream infections are associated with the use of central venous catheters (Rodrigues, Gomes et al. 2019). Medical interventions that compromise physiological barrier functions, such as implantation of medical devices, administration of parenteral nutrition, use of central venosus catheters, abdominal surgery, and broad-spectrum antibiotic therapy can further predispose patients to disseminated *Candida* infections (Polke et al. 2015; Pappas et al. 2018; Atiencia-Carrera et al. 2022; Gander-Bui et al. 2023).

As silicone materials are highly permissive to microbial biofilm formation, significant research efforts have focused on modifying silicone to improve its resistance to microbial colonization and extent the lifespan of silicone medical devices. Vaterrodt et al. introduced an innovative strategy for coating catheter materials using a layer-by-layer assembly of three newly synthesized functional polymeric building blocks, which displayed both antifouling and antimicrobial properties, leading to reduced biofilm formation and infection rates (Vaterrodt et al. 2016). Other studies have demonstrated that silicones incorporating 2% or 5% cationic biocides, specifically 1-dodecyl-3-methylimidazolium tetrafluoroborate and polyhexamethylene guanidine dodecylbenzenesulfonate, exhibit strong antimicrobial activity and excellent leaching resistance, identifying them as promising candidates for the development of safer, biofilm-resistant medical devices (Ghamrawi et al. 2017).

Two independent strategies for modifying silicone materials have shown considerable promise for enhancing resistance to microbial colonization and prolonging device longevity. The first approach, surface fluorination, has been shown to decrease surface energy, reduces wettability, and enhances the chemical resistance of silicone. Studies have demonstrated that fluorinated silicone surfaces exhibit significantly increased hydrophobicity, enhanced chemical stability, and reduced protein and cell adhesion, all of which contribute to reduced biofilm formation. These properties are attributed to the high electronegativity and low polarizability of C–F bonds, which produce exceptionally low surface energy and limit bio molecular interactions at the interface (Shan, An et al. 2017, Cardoso et al. 2018; Xing et al. 2024). The second promising approach involves the covalent incorporation of quaternary ammonium salts (QAS) into the silicone matrix. QAS exert their antimicrobial effect through electrostatic attraction between the positively charged ammonium groups and the negatively charged bacterial cell membrane, followed by insertion of hydrophobic alkyl chains into the lipid bilayer. This disrupts membrane integrity and ultimately causes cell death (Obłak and Gamian 2010; Liang et al. 2024). Unlike low-molecular-weight biocides, immobilized QAS coatings provide long-lasting contact-dependent antimicrobial activity and high resistance to active substance leaching (Fortuniak et al. 2011; Pan et al. 2024). Consequently, they can effectively inhibit the colonization of bacteria and yeasts, forming a robust protective barrier on silicone-based medical devices, including VPs.

Literature analysis indicates that fluorination and QAS functionalization are two complementary yet distinct approaches for silicone modification. Fluorination primarily focuses on enhancing chemical resistance and surface properties, while QAS functionalization is designed to impart durable antimicrobial activity. Despite extensive research on each technique individually, a systematic comparative study examining their respective effects on the structural and performance properties of silicones is lacking. This gap in knowledge underscores the relevance and necessity of our research.

Our work contributes to the development of silicone-based medical devices by aiming to extend their lifespan and improve patient safety during use. For this study, we selected three materials for investigation: pure silicone (SI), silicone functionalized with quaternary ammonium salts (SI + HEX), and fluorinated silicone (SI + F).

## Materials and methods

### Materials

Hexane (99%), N,N-dimethylhexadecylamine (≥ 95%), hydrochloric acid solution (37%), 3-chloropropyltrimethoxysilane (≥ 97%), and methyltrimethoxysilane (98%) were purchased from Merck. 1,1,3,3-Tetramethyl-1,3-divinyldisiloxane (98%) and 1,1,1,3,3,3-hexamethyldisiloxane (98%) were obtained from ABCR GmbH. SILPURAN® 6600/60 A/B was supplied by Wacker Chemie AG. All chemicals were used as received without further purification. Water was freshly deionized prior to use.

### Synthesis of functionalized MT siloxane resin

**Fig. 1:**
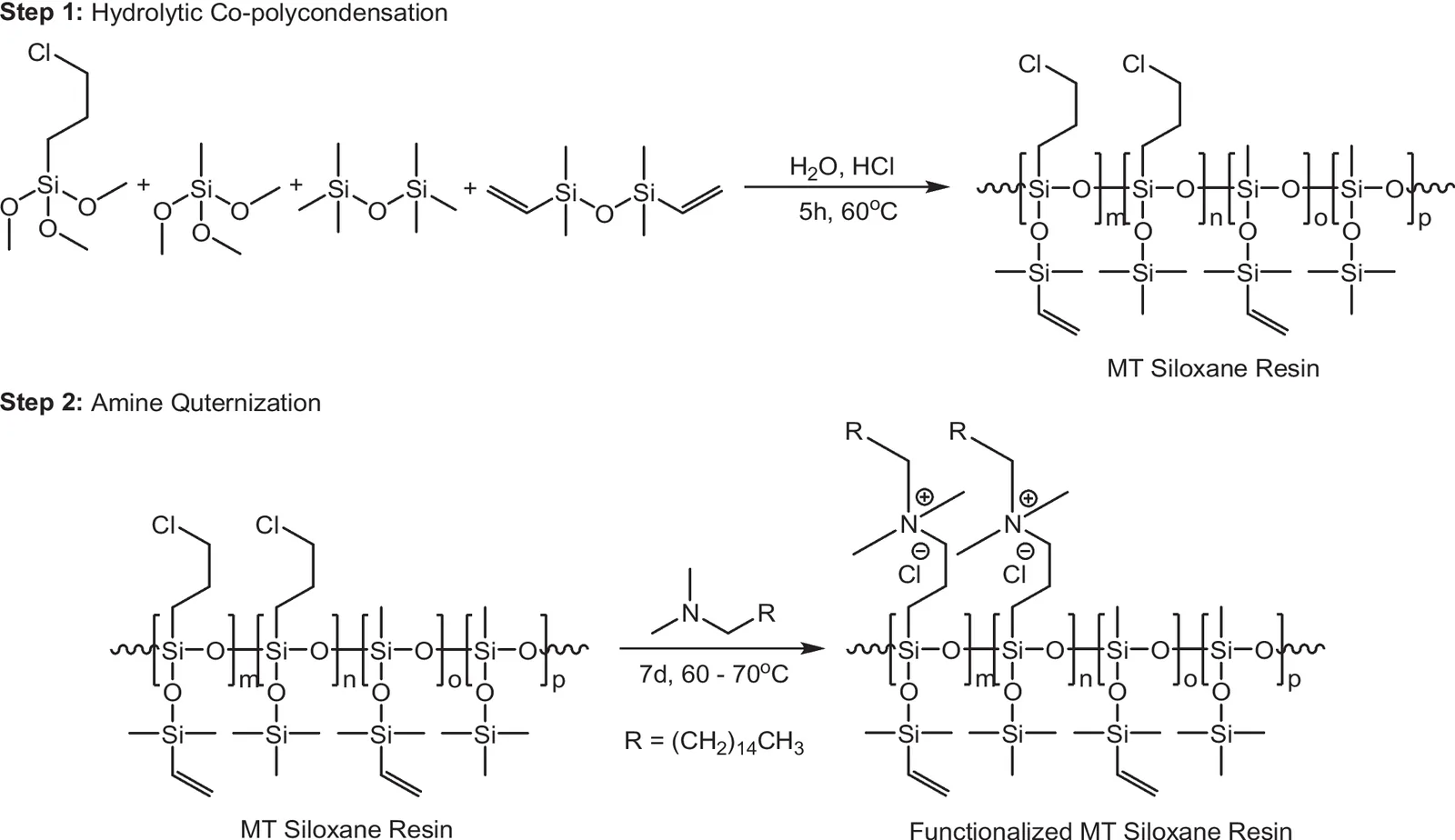
Schematic representation of the synthesis and functionalization of MT siloxane resin

The synthesis of MT siloxane resin, employed as an additive in LSR blends to confer antimicrobial properties, was carried out in two steps. Initially, the resin was prepared via acid-catalyzed hydrolytic co-condensation of selected organosilicon monomers, yielding MT siloxane resin functionalized with 3-chloropropyl and vinyl groups. In the second step, the resin was further modified through quaternization using N,N-dimethylalkylamine. The synthetic route is illustrated in Fig. 1.

Initially, 3-chloropropyltrimethoxysilane (21.28 g), methyltrimethoxysilane (15.7 g), 1,1,3,3-tetramethyl-1,3-divinyldisiloxane (1.0 g), and 1,1,1,3,3,3-hexamethyldisiloxane (17.84 g) were placed in a reactor equipped with a magnetic stirrer, condenser, thermometer, and heating jacket. A solution of concentrated HCl (4.84 g) in deionized water (9.52 g) was added in a single portion to the mixture under stirring, resulting in a rapid increase in temperature and viscosity. The reaction was carried out for 5 h at 60 °C under solvent-free conditions. Upon cooling to room temperature, the crude product was dissolved in 100 mL of hexane and extracted with three portions of deionized water (100 mL each) to remove methanol and residual HCl. The organic phase containing the MT siloxane resin was evaporated to remove any remaining hexane, methanol, and residual unreacted substrates. The resulting MT siloxane resin (38.66 g, 97.2% yield) was characterized by FT-IR spectroscopy to confirm its structure and determine the content of 3-chloropropyl and vinyl groups, and by GPC to evaluate its molecular weight.

For the second step, 30 g of the synthesized MT siloxane resin (Mn = 1088 g/mol, Mw = 1320 g/mol), containing 3-chloropropyl (0.00234 mol/g) and vinyl (0.000205 mol/g) groups, was combined with N,N-dimethylhexadecylamine (18.9 g) in a reactor equipped with a magnetic stirrer and heating jacket. The system was sealed and heated to 60–70 °C under solvent-free conditions for 7 days with continuous stirring, during which the viscosity of the mixture gradually increased. Upon completion, the reaction mixture was cooled to room temperature and analyzed by FT-IR spectroscopy to confirm successful quaternary ammonium functionalization.

### Preparation of the master batch additive for the LSR mixture

To ensure homogeneous dispersion of the functionalized resin within the injection-molded elements, a masterbatch of component B of the LSR (Wacker SILPURAN® 6600/60 A/B) containing 20 wt% of the functionalized resin was prepared. This was achieved by mechanically mixing 160 g of component B with 40 g of the functionalized MT siloxane resin for 1 h to obtain a uniform paste. The masterbatch was then introduced into the injection mold together with components A and B of the LSR via the pigment line, in an amount sufficient to achieve a final content of 1 wt% functionalized siloxane resin in the molded product.

### Fourier transform infrared (FT-IR) spectroscopy

FT-IR spectra were recorded using a BRUKER Tensor 27 Fourier transform spectrophotometer equipped with a Golden Gate single-reflection diamond ATR unit. For each measurement, 16 scans were collected at a resolution of 4 cm⁻^1^, over the spectral range of 4000–500 cm⁻^1^.

### Gel permeation chromatography (GPC)

GPC analyses were performed using a chromatographic system consisting of a Waters 2695 separations module, a Waters 2414 RI detector, and a set of three serially connected 7.8 × 300 mm columns (Waters Styragel HR1, HR2, and HR4). Tetrahydrofuran (THF) was used as the mobile phase at a flow rate of 0.6 mL/min. The column oven was maintained at 35 °C and the detector at 40 °C. The molecular weights (Mn, Mw) and polydispersity index (PDI) were calculated based on a calibration curve using polystyrene standards (Shodex) in the range of 1.31 × 10^3^ to 5.91 × 10^5^ g/mol.

### Assessment of biofilm mass – crystal violet (CV) staining

VPs after 3 months of patient use and new VPs (control group) were cut into four equal fragments per group. Each fragment was weighed and transferred into a 96-well plate. The samples were rinsed with NaCl and fixed with 90% ethanol for 10 min, then allowed to air-dry. Subsequently, the samples were stained with 0.5% crystal violet (Argenta) for 10 min, rinsed three times with distilled water, and left to dry. The stained samples were then incubated with 75% ethanol, and 100 µL of the resulting suspension was transferred to empty wells of a 96-well plate. Absorbance was measured at 590 nm using a Tecan Spark spectrophotometer (Spark Control Magellan V2.2, Austria).

### Fungal strains used for in vitro biofilm studies

As shown in our previous study (Spalek et al. 2020), the main microorganisms present in the biofilm formed on voice prostheses and responsible for their damage and deformation were *Candida krusei, Candida albicans, Candida glabrata*, and *Candida tropicalis.* Therefore, in this study, we used four clinical fungal strains (one isolate from each of the above-mentioned species) to form the biofilm in vitro on the tested materials. These strains were part of a collection of frozen strains from damaged Provox VPs obtained from laryngectomy patients at Holy Cross Cancer Center. The exclusive use of *Candida* strains in assessing the impact of microorganisms on the silicone materials tested was also dictated by previous studies indicating that the ability of *Candida* spp. to damage and deform silicone in voice prostheses stems primarily from their ability to form hyphae and penetrate deep into these polymers. Although bacteria present in mixed biofilms facilitate fungal colonization, they are primarily microbial colonizers of the oral cavity and are less significant in causing potential damage. *Candida* strains were cultured on Sabouraud agar (Thermo Fisher Scientific), identified using routine laboratory procedures (colony morphology, Gram staining, Vitek 2 system (bioMerieux)), and then frozen at − 70 °C in a cryobank system (Mast Diagnostics). Before the experiment, strains were reactivated on Sabouraud agar. To form a biofilm, VP (SI, SI + HEX, or SI + F) were placed in a sterile pharynx model and covered with culture medium (CM) containing a mixture of fungal strains. A suspension of 1 McFarland density was prepared from each strain in TSB broth and mixed with equal volumes (Thermo Fisher Scientific). Samples were incubated for 48 h at 37 °C and then transferred to a TEF simulator for 14 days, during which they were continuously perfused with TSB broth. Experiments using the TEF simulator were performed according to the procedure described in our previous studies, which also described the device design in detail (Okła et al. 2026). After incubation, the VP was gently rinsed with 0.9% NaCl and fixed in 10% formaldehyde.

*Microscopic observations—confocal microscopy and scanning electron microscopy*Microscopic observations of the surface topography of new VPs, VPs removed from patients, and VPs incubated in the trachea-esophageal fistula (TEF) simulator were performed using confocal and scanning electron microscopy (SEM). New VPs were used as controls. When required, the VPs fragments were mechanically cleaned to visualize the underlying surface under SEM. Surface texture was additionally analyzed using a Leica DCM8 confocal microscope at 20 × magnification in confocal mode. Analysis included optical images, 2D surface maps, 3D axonometric images, and surface profiles, covering a total area of 1.44 mm^2^. For in vitro biofilm formation, VPs (SI, SI + HEX, or SI + F) were placed in a sterile throat model and covered with culture medium (CM). The samples were incubated for 48 h at 37 °C, then transferred to the TEF simulator for 14 days, during which they were continuously perfused with CM. After incubation, the VPs were gently rinsed with 0.9% NaCl and fixed in 10% formaldehyde. Prior to SEM analysis using a Phenom XL microscope, the samples were dehydrated through a graded ethanol series (30, 50, 70, 80, 90, 96, and 100%; 10 min each), air-dried, and then sputter-coated with a thin layer of gold to ensure conductivity. Imaging was performed at an accelerating voltage of 5 kV using secondary electron (SE) detection mode to optimize image quality while minimizing sample damage. The surfaces were observed at 255 × magnification.

### Atomic force microscope studies

Microscopic evaluation of VPs made of SI, SI + HEX, or SI + F was performed using the NanoWizard 4 BioScience atomic force microscope (AFM – JPK/Bruker, MA, USA). To evaluate the surface topography of VPs after 72 h of *Candida albicans* biofilm growth, VP fragments were incubated for 72 h at 37 °C in sterile plates containing a suspension of *Candida albicans* cells. After incubation, the VP fragments were gently rinsed with PBS to remove planktonic cells and examined using the AFM microscope. To minimize the influence of lateral forces associated with contact mode scanning, Quantitative Imaging (QI) mode (JPK QI™ mode) was employed. QI maps from VPs post-incubation were acquired using Bruker MSCT-E AFM probes with a nominal spring constant of 0.1 N/m and a measured spring constant in the range of 0.10—0.17 N/m (determined using the thermal tune method). Topography maps of 50 × 50 µm and 20 × 20 µm were recorded with a resolution of 128 pixels per line under wet conditions. Using the same type of AFM probes, further VP surfaces characterization was performed after biofilm removal. QI maps (10 × 10 µm) were utilized to quantify the physicochemical properties of the VP surfaces. QI Height Mode was applied to visualize the topography and determine average surface roughness values (Ra); QI Slope Mode to assess surface stiffness; QI Adhesion Mode to measure adhesion forces between the AFM probe and VP surfaces. The calculated parameters characterizing the VP surfaces using AFM are shown in Sect. 3.4.

### Cell culture studies

The NIH 3T3 fibroblast cell line from ATCC (CRL-1658) was used. Fibroblasts were cultured in DMEM (Dulbecco’s Modified Eagle Medium) supplemented with 10% FBS, 2.5 mM L-glutamine, non-essential amino acids (NEAA) and penicillin–streptomycin solution. The flasks were incubated at 37 °C in 5% CO_2_. Equal pieces of the tested VP made of SI, SI + HEX, or SI + F were placed in culture medium (200 mg/ml) and incubated for 24 h at 37 °C. After the incubation time, the culture medium was filtered using a sterile 0.2 μm syringe filter (Corning; Germany), and then 500 μL of the extract was mixed with the fibroblast suspension and placed in 24-well plates. Cell cultures were placed inside the chamber equipped with an ASI x/y/z stage (BioVision Technologies). Cell area and circularity index were determined from optical images collected with 10 × air lenses and phase contrast using a Hamamatsu camera on a Leica DMIRE2 inverted microscope (Leica). For each condition, a minimum of 5 (10 ×) images were acquired, and 50–100 cells per condition were analyzed. Cell area was calculated using Fiji software by tracing cell peripheries (Fiji Software, NIH). Only single cells were taken into account.

### Assessment of hemolytic properties and cytokines release

VP made of SI, SI + HEX, or SI + F were cut into small, equal fragments of a defined surface area to ensure reproducibility of exposure. Human blood was collected into heparinized tubes. Whole blood samples were then incubated with VP fragments under sterile conditions. Incubation was performed for 24 h at 37 °C. During incubation at 1, 6, 10 and 24 h time points, the percentage of hemolysis of whole blood was determined. Hemolysis was calculated based on a standard hemoglobin calibration curve.

In order to more precisely explore the inflammatory profile of different types of silicone VP's a Proteome Profiler Human Cytokine Array Kit (R&D; #ARY005B) was used. The array simultaneously detects 36 human cytokines, chemokines, and acute phase proteins including C5a, IL-4, IL-27, CD40 Ligand, IL-5, IL-32 alpha, G-CSF, IL-6, CXCL10/IP-10, GM-CSF, IL-8, CXCL11/I-TAC, CXCL1/GRO alpha, IL-10, CCL2/MCP-1, CCL1/I-309, IL-12 p70, MIF, ICAM-1, IL-13, MIP-1 alpha/MIP-1 beta, IFN-gamma, IL-16, CCL5/RANTES, IL-1 alpha, IL-17, CXCL12/SDF-1, IL-1 beta, IL-17E, Serpin E1/PAI-1, IL-1ra, IL-18, TNF-alpha, IL-2 IL-21, and TREM-1. Samples, reagents, and immunoassay procedures were prepared and performed according to the manufacturer’s instructions. Results were normalized to the control (samples without prosthesis fragments) to compare cytokine expression between groups.

### Mechanical tests—tensile strength, elongation at break and Shore hardness

**Fig. 2:**
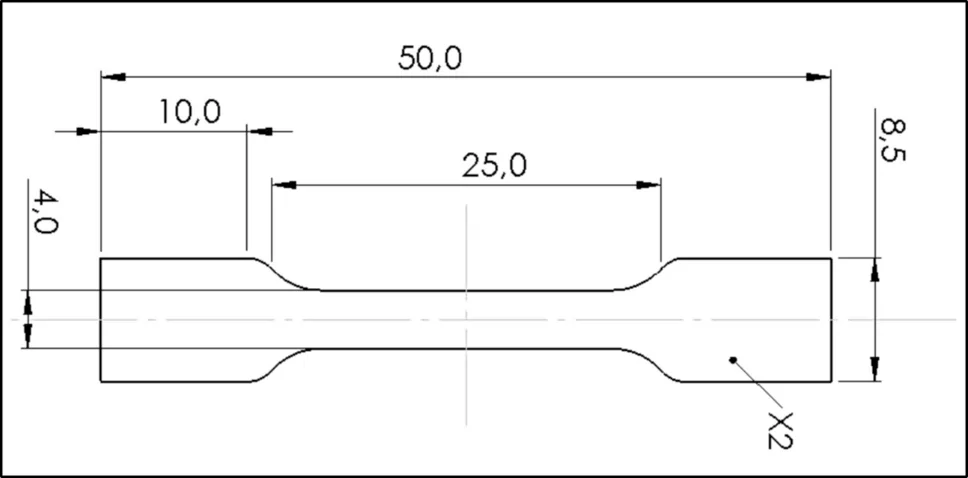
View of the sample used for tensile strength tests

Strength parameters were tested using a Galdabini QUASAR testing machine. The tests were performed in accordance with the PN-EN ISO 527–1:2020–01. Tensile strength and elongation at break were analyzed during the tests. The test parameters are presented in Table S2, and the sample view is shown in Fig. 2. The test results presented are average values calculated on the basis of 10 measurements.

The elongation at break was calculated using the formula:

$$\mathrm{A}=\frac{({\mathrm{L}}_{\mathrm{u}}-{\mathrm{L}}_{\mathrm{o}})}{{\mathrm{L}}_{\mathrm{o}}}*100\mathrm{\%}$$

where:

$$\mathrm{A}-\mathrm{p}\mathrm{e}\mathrm{r}\mathrm{c}\mathrm{e}\mathrm{n}\mathrm{t}\mathrm{a}\mathrm{g}\mathrm{e} \mathrm{e}\mathrm{l}\mathrm{o}\mathrm{n}\mathrm{g}\mathrm{a}\mathrm{t}\mathrm{i}\mathrm{o}\mathrm{n} \mathrm{a}\mathrm{f}\mathrm{t}\mathrm{e}\mathrm{r} \mathrm{b}\mathrm{r}\mathrm{e}\mathrm{a}\mathrm{k}, \mathrm{\%}$$

$${\mathrm{L}}_{\mathrm{u}}-\mathrm{l}\mathrm{e}\mathrm{n}\mathrm{g}\mathrm{t}\mathrm{h} \mathrm{o}\mathrm{f} \mathrm{t}\mathrm{h}\mathrm{e} \mathrm{s}\mathrm{p}\mathrm{e}\mathrm{c}\mathrm{i}\mathrm{m}\mathrm{e}\mathrm{n} \mathrm{a}\mathrm{f}\mathrm{t}\mathrm{e}\mathrm{r} \mathrm{b}\mathrm{r}\mathrm{e}\mathrm{a}\mathrm{k} \left(\mathrm{i}.\mathrm{e}. \mathrm{f}\mathrm{i}\mathrm{n}\mathrm{a}\mathrm{l} \mathrm{m}\mathrm{e}\mathrm{a}\mathrm{s}\mathrm{u}\mathrm{r}\mathrm{e}\mathrm{d} \mathrm{l}\mathrm{e}\mathrm{n}\mathrm{g}\mathrm{t}\mathrm{h}\right), \mathrm{m}\mathrm{m}$$

$${\mathrm{L}}_{0}-\mathrm{i}\mathrm{n}\mathrm{i}\mathrm{t}\mathrm{i}\mathrm{a}\mathrm{l} \mathrm{l}\mathrm{e}\mathrm{n}\mathrm{g}\mathrm{t}\mathrm{h} \mathrm{o}\mathrm{f} \mathrm{t}\mathrm{h}\mathrm{e} \mathrm{s}\mathrm{p}\mathrm{e}\mathrm{c}\mathrm{i}\mathrm{m}\mathrm{e}\mathrm{n}, \mathrm{m}\mathrm{m}$$

The hardness of the materials was determined using the Shore’a A method on a Hildebrand device. During the test, the durometer is placed perpendicular to the surface of the specimen and loaded with a force of 1 kg for 1 s. The mean value of the Shore A hardness was determined based on 10 measurement series. The tests were performed in accordance with PN-EN ISO 868: 2005.

### Wettability measurements and the surface free energy (SFE)

Assessment of this properties were carried out using a sessile drop method on an Ossila goniometer (Sheffield, UK), following the procedures outlined in ISO 25178–607:2019. Three liquids with different surface tensions were used: water (72.75 mN/m, Milli-Q), acetone (23.24 mN/m, Chempur, Poland), and ethanol (22.31 mN/m, Chempur, Poland). A 5 µl droplet of each liquid was dispensed onto the surface, and the contact angle was measured immediately. For each liquid, measurements were performed in triplicate, and the average of the left and right contact angles was recorded.

SFE was estimated using the Zisman method. This involved measuring the contact angles of a series of liquids with known surface tensions (water: 72.75 mN/m, acetone: 23.24 mN/m, and ethanol 22.31 mN/m) on the tested solid surface. The cosine of each contact angle was plotted against the corresponding liquid surface tension. A linear fit was applied to the data points, and the line was extrapolated to cos θ = 1. The surface tension value at this intercept, where the contact angle theoretically becomes zero, is known as the critical surface tension. According to the Zisman theory, this critical surface tension was taken as an approximation of the surface free energy of the solid.

### Thermogravimetry (TGA)

TGA tests were carried out using a Q500 thermogravimetric analyzer (TA Instruments, New Castle, DE, USA) in a temperature range from 25 to 1000 °C and a nitrogen atmosphere, with heating rate ($\mathrm{k}$) values of 5, 10, and 20 °C/min. Special attention was paid to the temperature of thermal decomposition (T_DS_; taken as 1% and 5% of the weight loss of the sample). The Kissinger method was used to determine the activation energy of the thermal decomposition of the tested materials. This method is based on the dependence of the temperature value T_max._ (corresponding to the maximum DTG signal) to the heating rate $\mathrm{k}$:

$$\mathrm{l}\mathrm{n}(\frac{\mathrm{k}}{{\mathrm{T}}_{\mathrm{m}\mathrm{a}\mathrm{x}}^{2}})=-\frac{{\mathrm{E}}_{\mathrm{a}}}{\mathrm{R}}\cdot \frac{1}{{\mathrm{T}}_{\mathrm{m}\mathrm{a}\mathrm{x}}}+\mathrm{c}\mathrm{o}\mathrm{n}\mathrm{s}\mathrm{t}.$$

As the heating rate increases, the temperature of the maximum intensity of the DTG signal also increases. The Kissinger method is based on presenting the obtained T_max._ values in the configuration. The directional coefficient of the obtained straight line corresponds to the value (where $\mathrm{E}\mathrm{a}$ is the activation energy, and $\mathrm{R}$ is a gas constant equal to 8.314 J/mol·K).

### Statistical analysis

Results are reported as mean ± SD for all experimental replicates. Differences between treated samples and the controls were determined using a one-way ANOVA followed by Tukey’s multiple comparisons test, with p < 0.05 considered statistically significant (Figs. 6, 11, and 12). A separate linear regression model was run for each variable, and significance was defined as p < 0.05 (Figs. 9 and 10). A multivariable linear regression model that included all variables revealed a significant interaction among SI, SI + HEX, SI + F, and the control condition (Fig. 9). In contrast, a corresponding multivariable model showed no significant interaction among SI, SI + HEX, and SI + F (Fig. 10A). All statistical analyses were conducted using BioRender’s online software.

## Results

### FT-IR analysis of MT siloxane resins

**Fig. 3:**
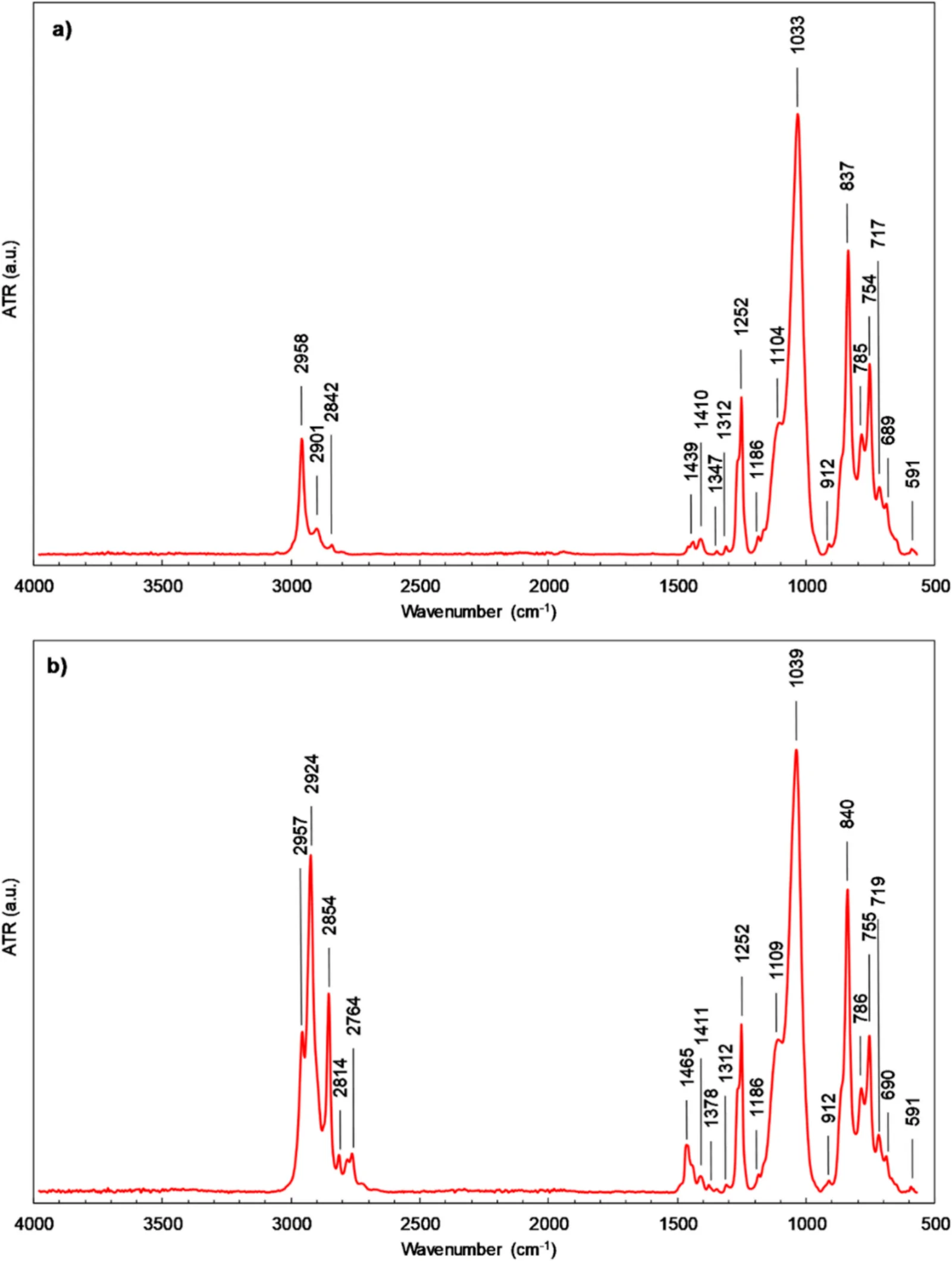
FT-IR spectra of (**A**) MT siloxane resin and (**B**) functionalized MT siloxane resin

Both the MT siloxane resin obtained in the first synthesis step and the resin following functionalization were analyzed by FT-IR spectroscopy. The FT-IR spectra were also used to quantify the reactive 3-chloropropyl and vinyl groups present in the MT siloxane resin. The FT-IR spectra of the MT siloxane resin and the functionalized resin are presented in Fig. 3 A and B, respectively.

The absorption pattern and band intensities in the spectrum of the MT siloxane resin (Fig. 3A) confirm the assumed structure and composition of the resin. The presence of vinyl groups is evidenced by characteristic absorption bands at 3053, 1596, 1410, 959, and 784 cm⁻^1^, corresponding to stretching and bending vibrations of C–H and C = C bonds. Bands with maxima at 1347, 1311, and 911 cm⁻^1^ are characteristic of C–Cl stretching and bending vibrations from 3-chloropropyl groups.

The presence of alkyl chains of 3-chloropropyl groups is confirmed by absorption bands at 2958, 2900, and 2842 cm⁻^1^ (C–H stretching in methyl groups) and at 1186 and 1166 cm⁻^1^ (C–C skeletal vibrations). Additionally, bands at approximately 1250 and 750 cm⁻^1^ correspond to C–H stretching vibrations in dimethyl and trimethylsilyl groups, while an intense band at 1033 cm⁻^1^ arises from asymmetric Si–O–Si stretching vibrations in the siloxane backbone. The split band near 1250 cm⁻^1^ results from overlapping contributions of dimethyl, trimethylsilyl, and methylsilyl groups.

The content of functional groups was determined by integrating the areas of the characteristic absorption bands and comparing them with those of reference substances. The content of vinyl groups was determined as 0.205 mmol/g (based on the band at 1596 cm⁻^1^), while the content of 3-chloropropyl groups was 2.34 mmol/g (based on the band at 1347 cm⁻^1^). 1,1,3,3-Tetramethyl-1,3-divinyldisiloxane and 3-chloropropyltrimethoxysilane were used as reference compounds for calibration. The determined values correlate well with the stoichiometry of the starting materials and confirm the expected structure of the resin.

Compared to the initial MT resin spectrum (Fig. 3A), the FT-IR spectrum of the functionalized MT siloxane resin (Fig. 3B) exhibits new intense bands in the 3000–2750 cm⁻^1^ range, corresponding to C–H stretching vibrations of methylene groups in the alkyl chains of N,N-dimethylhexadecylamine. A new, low-intensity band at approximately 3400 cm⁻^1^, characteristic of quaternary ammonium salt derivatives, further confirms successful quaternization. Importantly, the retention of the Si–O–Si band at 1038 cm⁻^1^ indicates that the siloxane backbone remained intact during the functionalization process.

### Biofilm mass on VP replaced after 3 months of use

**Fig. 4:**
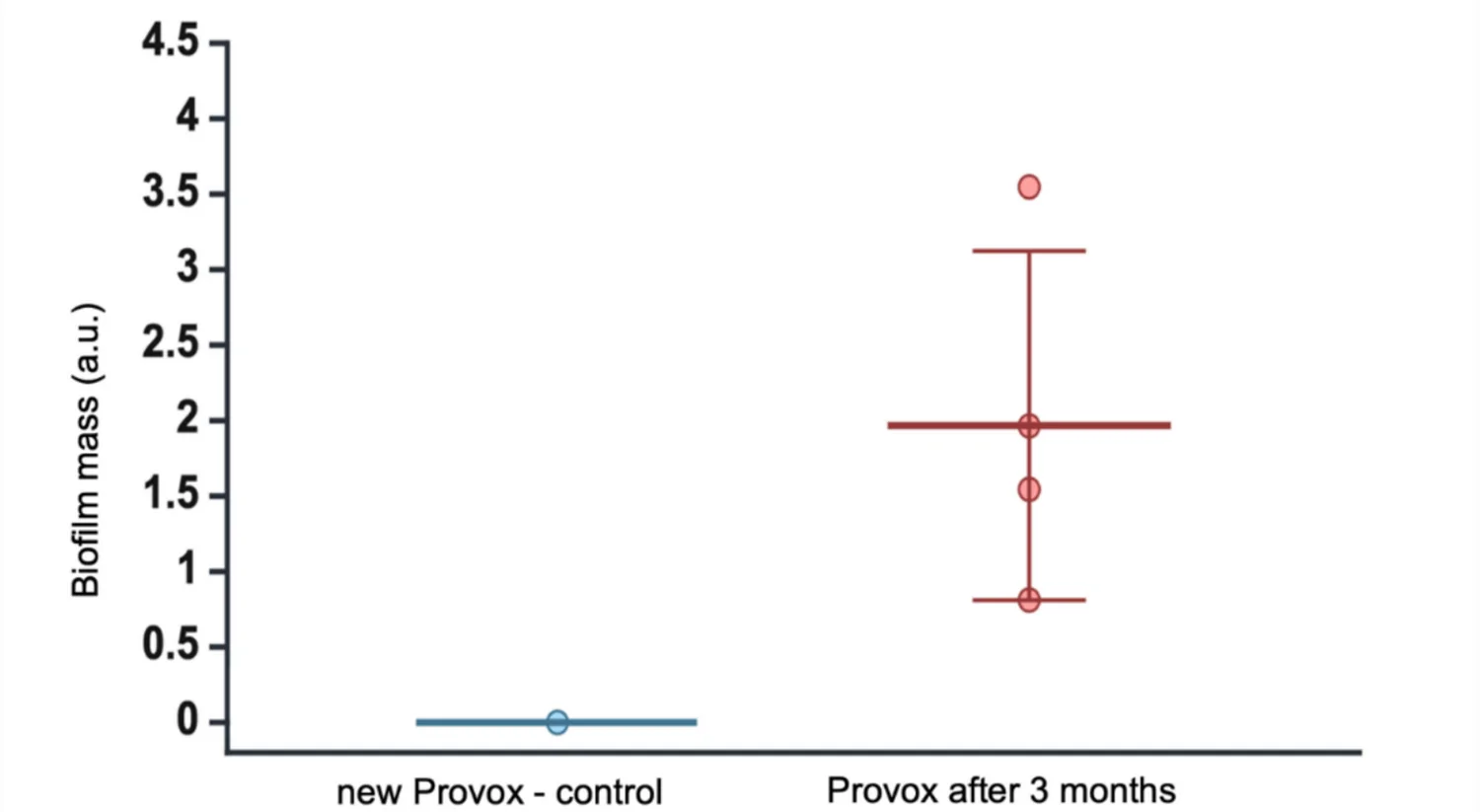
Relative biofilm mass on Provox voice prostheses (n = 4) after 3 months of use, expressed as the fold increase relative to a new Provox device. Biofilm quantification was performed using crystal violet staining

After three months of Provox VP use, a substantial amount of biofilm was observed on the device surface (Fig. 4). Moreso, the median biofilm load varied between patients in the “Provox after 3 months” group, indicating individual differences in biofilm formation that may be influenced by environmental factors such as diet (Santacroce et al. 2023).

### Microscopic observations

Surface topography analysis was performed using microscopic images of the Provox VP in an unused state and 3 months after implantation.

**Fig. 5:**
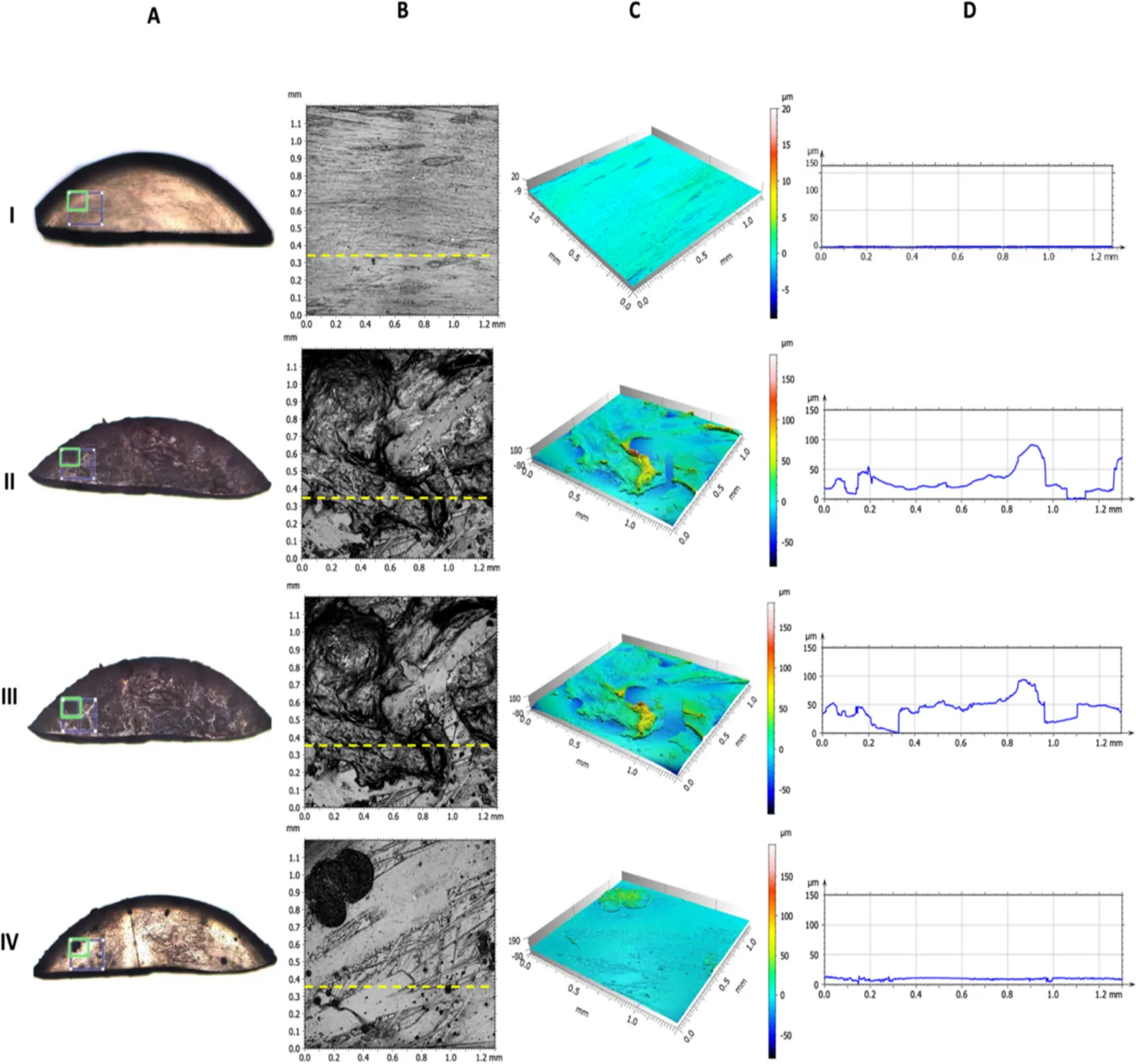
Microscopic imaging of a brand new Provox voice prosthesis (VP) and a VP removed for replacement after 3 months of use, showing biofilm deposition and subsequent cleaning steps. Panels: **I** – brand new VP (control) **II** – VP immediately after replacement; **III** – VP fixed with formalin; **IV**—VP after mechanical removal of biofilm. Panels **IA, IIA, IIIA, IVA**– magnification × 5 with marked area for next measurements. Panels **IB, IIB, IIIB, IVB** – magnification × 20 with the line of surface profile measurement. Panels **IC, IIC, IIIC, IVC –** 3D axonometric images. Panels **ID, IID, IIID, IVD** – profiles of the surface

Microscopic imaging of the new Provox VP confirms a smooth and uniform surface, showcased by both low and higher magnification images (Fig. 5, IA and IB). These reveal a homogenous texture and even surface within the marked measurement area. The 3D axonometric reconstruction (Fig. 5, IC) demonstrated a regular and continuous surface, while the surface-profile analysis (Fig. 5, ID) revealed only minimal deviations from a flat baseline, indicating very low surface roughness.

In contrast, the VP examined immediately after removal from the patient showed clear evidence of biofilm deposition. Low-magnification imaging (Fig. 5, IIA) revealed visible biofilm structures on the surface. The enlarged image (Fig. 5, IIB) confirmed heterogeneous and uneven distribution of the biofilm mass. These features corresponded with the 3D axonometric image (Fig. 5, IIC), which displayed pronounced irregularities and elevated regions associated with biofilm accumulation. The surface-profile plot (Fig. 5, IID) showed substantial deviations from a flat line, reflecting markedly increased surface roughness due to biofilm presence.

Formalin fixation of the explanted VP did not disrupt the characteristic biofilm structure. Imaging of the fixed sample (Fig. 5, IIA) showed a distinct, dense structure, similar to that observed prior to sample fixation. The magnified image (Fig. 5, IIIB) of the surface further confirmed an irregular, biofilm covered surface. The 3D axonometric reconstruction (Fig. 5, IIIC) shows irregularities and surface unevenness, and the corresponding profile (Fig. 5, IIID) showed large deviations from a flat line, consistent with high roughness.

**Fig. 6:**
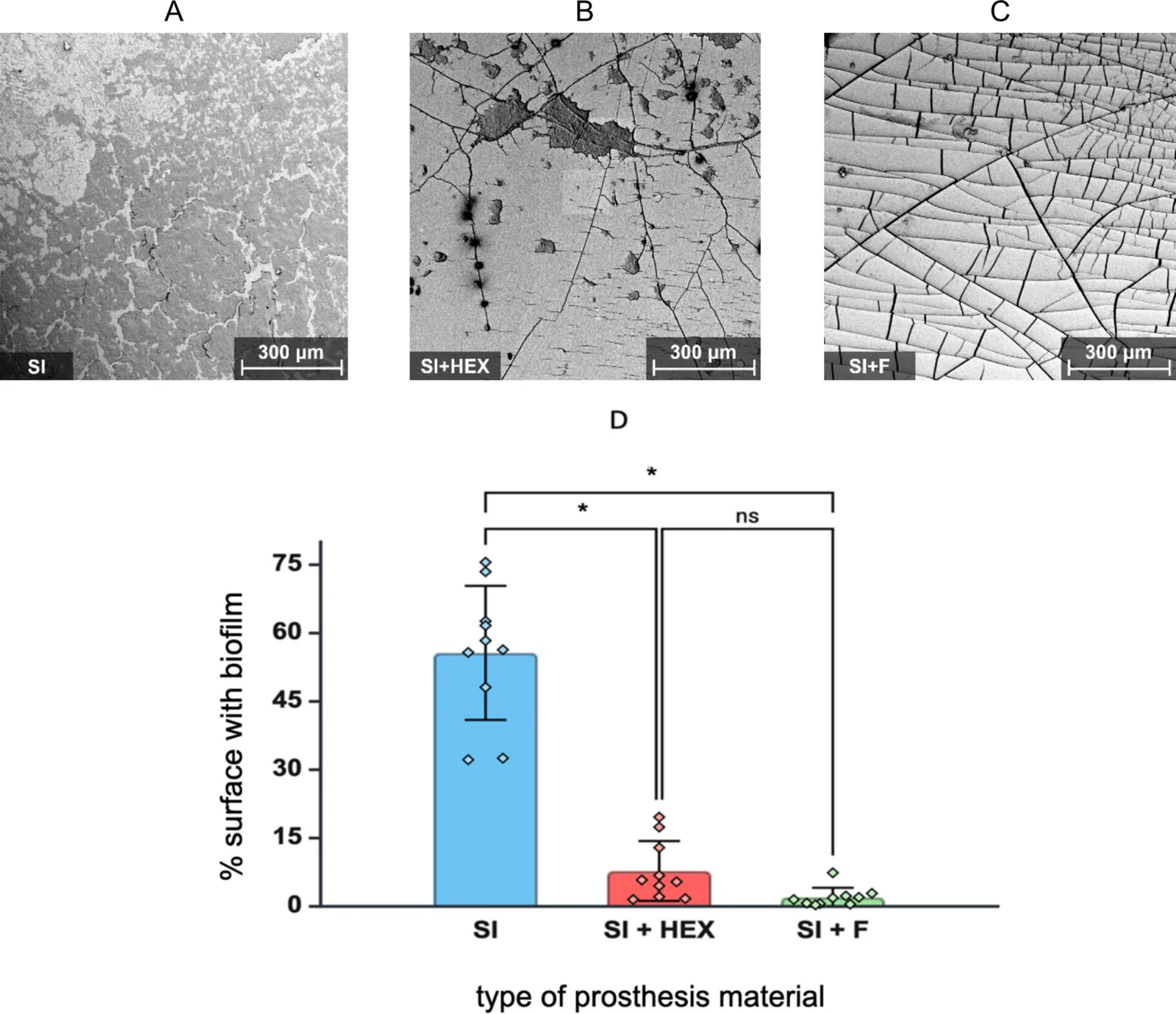
Voice prosthesis surface covered with biofilm after 48 h of incubation in a culture medium containing *Candida* cells and 14 days of growth in a TEF simulator (in vitro). SEM imaging: Panel **A**—SI, Panel **B**—SI + HEX and Panel **C**—fluorinated SI + F. Panel **D** shows average measurements (n = 10) and the mean and SD for each type of the prostheses. Scale bar = 300 µm. Magnification – 255x. Statistical analysis was evaluated by Tukey’s multiple comparison test. (*) denote statistically significant differences between groups (p < 0.05), ns—no significant difference

Mechanical cleaning following formalin fixation resulted in partial removal of the biofilm. Figure 5, IVA showed areas where the biofilm had been removed alongside regions where residues remained. Higher magnification imaging revealed a visibly smoother surface compared to the uncleaned samples, although some irregularities remained. The surface-profile analysis (Fig. 5, IVD) demonstrated smaller deviations relative to the untreated biofilm-covered samples (Panels II and III), indicating a measurable decrease in surface roughness following mechanical cleaning. Figure 6 presents the evaluation of biofilm development on the surfaces of the tested VPs made from silicone materials of different compositions (Panels A–C).

Analysis of biofilm formation on silicone surfaces revealed substantial differences among the materials tested. The SI silicone exhibited significant biofilm coverage (Fig. 6A–C), whereas both SI + HEX and SI + F showed markedly reduced biofilm growth. Quantitative assessment showed that SI had a significantly higher percentage of biofilm-coverage compared with both SI + HEX and SI + F (Fig. 6D). The difference between SI + HEX and SI + F appears less noticeable, although SI + HEX shows a slightly higher percentage of biofilm coverage.Statistical analysis confirmed significant differences in biofilm coverage between SI and either SI + HEX or SI + F, while no significant difference was observed between SI + HEX and SI + F.

### Characterization of tested VP surfaces using AFM

**Fig. 7:**
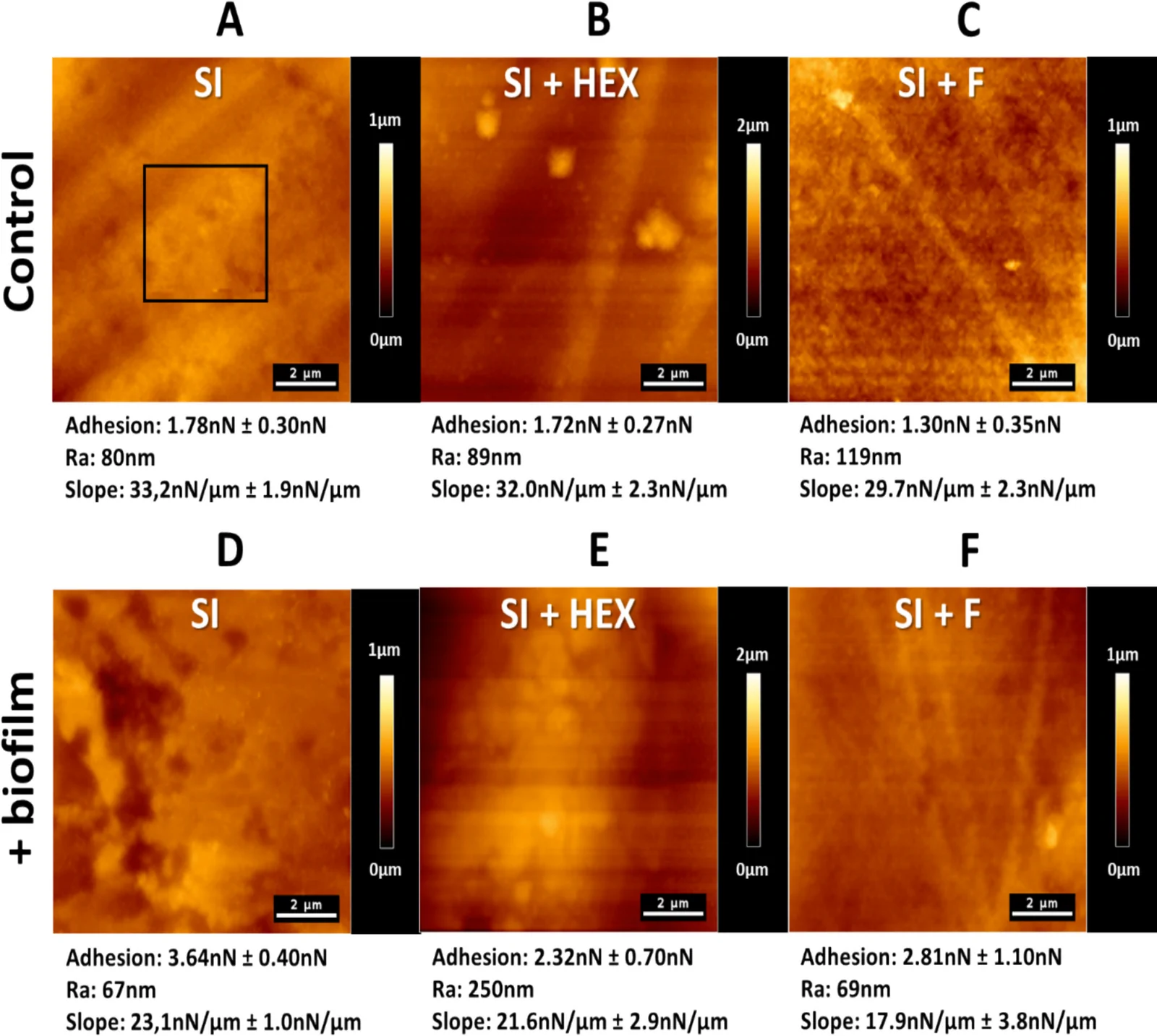
Characteristics of the surface of control VP (CT) and VPs after biofilm formation and removal. Panels **A-C**: control samples. Panels **D-F**: samples after biofilm removal; Panels **A**, **D**: silicone SI; Panels **B, E**: SI + HEX; Panels **C, F**: SI + H. Physical parameters characterizing the surface are provided below each panel

**Fig. 8:**
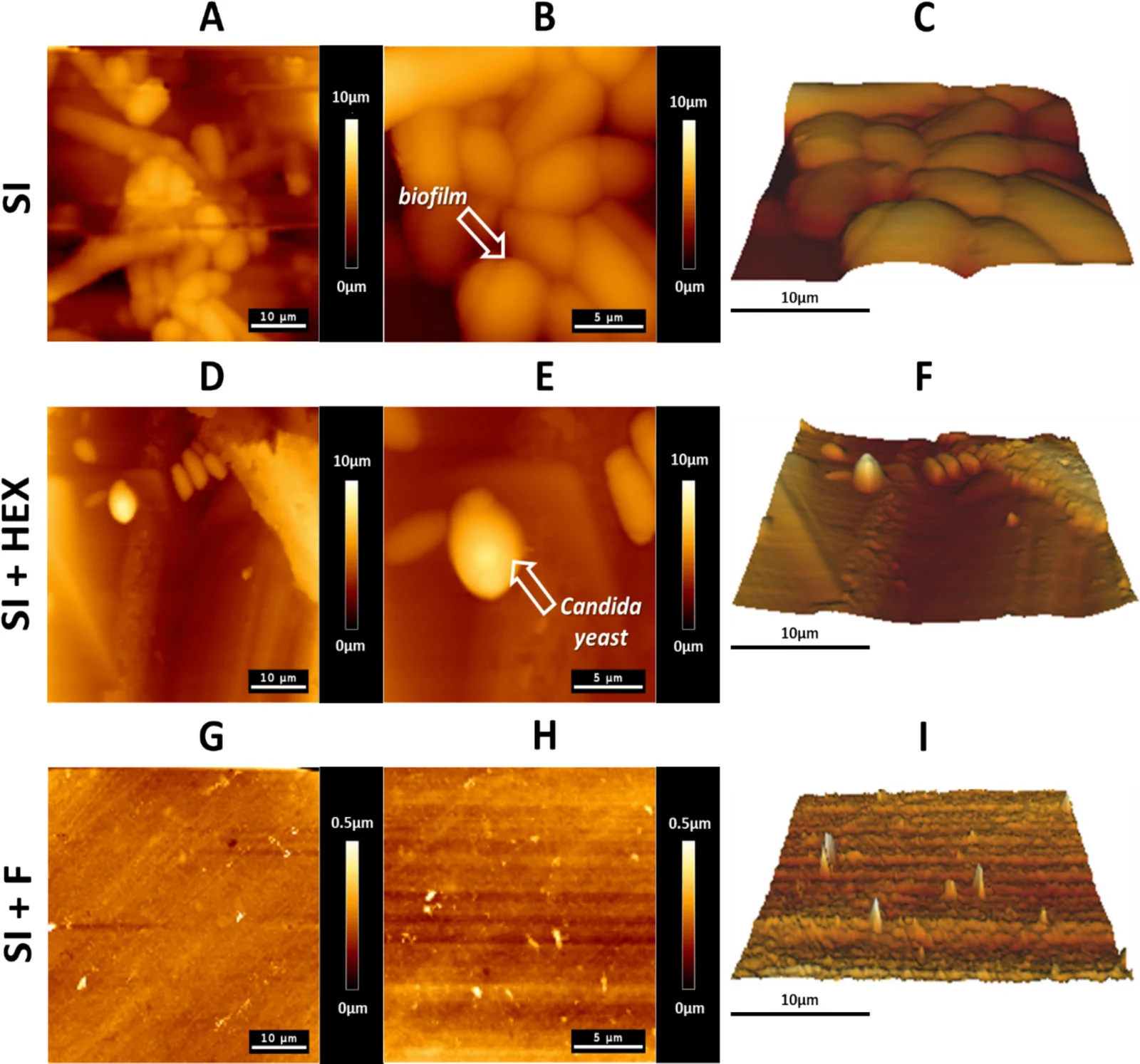
Surface topography of voice prostheses after 72 h of *Candida* biofilm growth. Panels **A-C**: SI; Panels **D-F**: SI + HEX; Panels **G-I**: SI + F

To estimate the impact of *Candida* biofilm formation on the surface properties of the tested polymers, atomic force microscopy was used to determine the physicochemical properties of the surface before and after *Candida* biofilm removal, including surface roughness (Ra parameter), surface stiffness (slope parameter), and adhesiveness (Figs. 7 and 8).

Measurements performed on the control polymers and on samples following biofilm removal demonstrated that biofilm formation substantially altered surface properties. All materials exhibited increased surface adhesiveness after biofilm exposure, with adhesion rising 104% for SI, 35% for SI + HEX, and 116% for SI + F. For SI and SI + F, the Ra parameter decreased after biofilm removal by 21% and 42%, respectively. In contrast, for SI + HEX, Ra increased by 180% after the biofilm was removed. In all tested materials, biofilm formation resulted in decreased surface stiffness by 30% for SI, 32.5% for SI + HEX, and 40% for SI + F. Among the materials, SI + F exhibited the greatest increase in adhesiveness and the most pronounced reduction in stiffness. These changes in surface characteristics may influence the durability of the prosthesis, however the modified materials show clear advantages with respect to biofilm resistance. Figure 8 shows the surface topography of the investigated materials after *Candida* biofilm formation. This revealed extensive biofilm coverage on SI, whereas the modified materials displayed markedly reduced biofilm deposition. For SI + HEX, only individual *Candida* cells were observed, while for SI + F, no yeast cells or biofilm were detected. These observations are consistent with the results of other microscopic analyses performed in this study.

The effectiveness of the tested materials in inhibiting biofilm growth was also confirmed by additional studies, when biofilm was stained with the Live/Dead viability kit (SYTO 9/propidium iodide) according to the manufacturer's protocol (Thermo Fisher Scientific, USA). The results of this study confirm the significant limitation of the growth of the *Candida* biofilm on the surface of silicone functionalized with both ammonium salts and fluorine as well as the lack of staining of potentially necrotic microorganism cells, which suggests their detachment or a much stronger inhibition of growth/adhesion than a direct fungicidal effect (**Figure S1**). This observation was further confirmed by a reduction in the number of cells colonizing the tested silicon (SI + HEX; SI + F) surface (**Figure S2**).

### Cell area and circularity index of fibroblast subjected to extract from tested VP

**Fig. 9:**
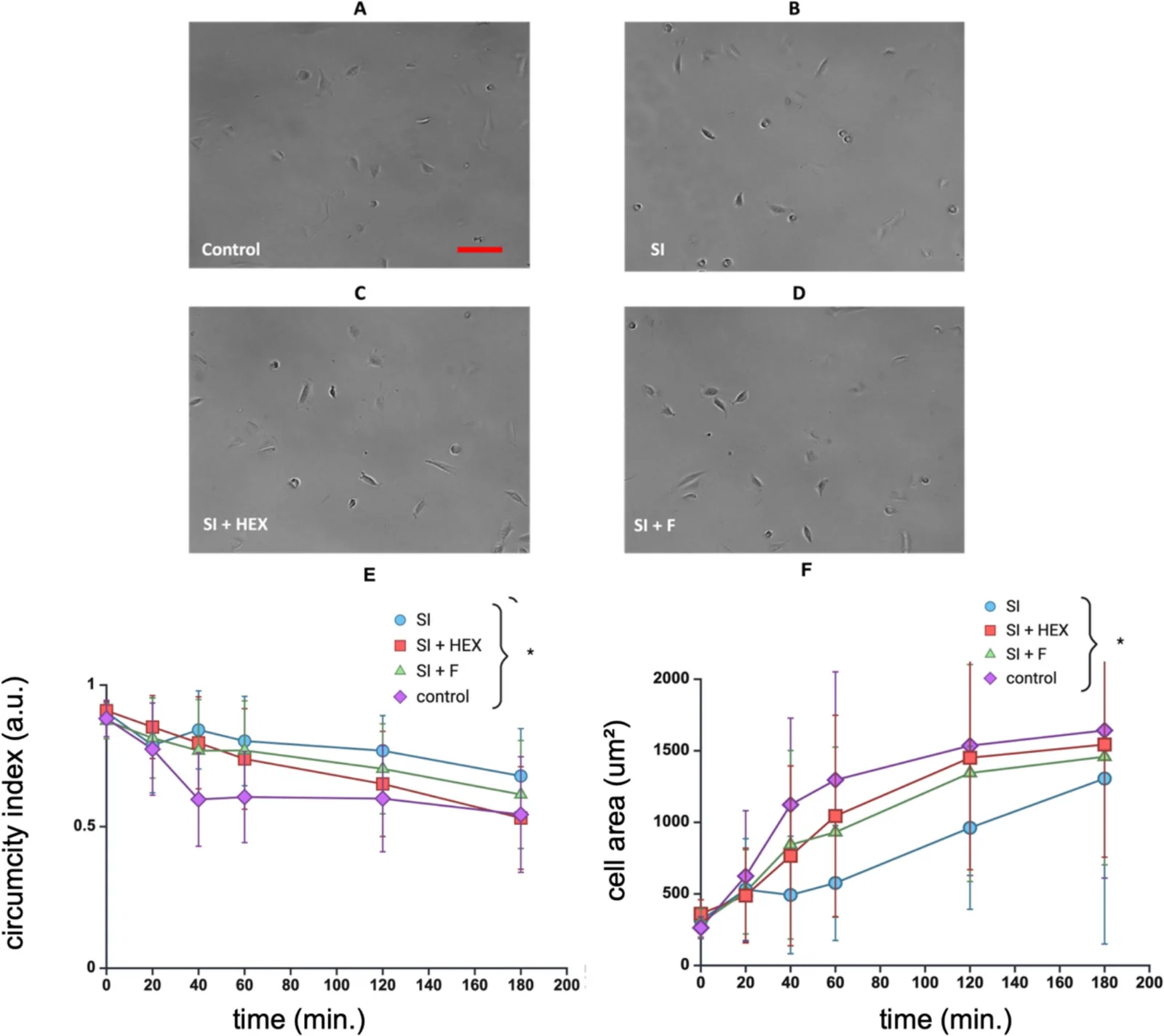
Morphological changes and circularity index of NIH 3T3 fibroblast. Panels: **(A)** morphology of fibroblasts growing on the surface of plastic cell culture plates—control (untreated), **(B)** with addition of extract from SI, **(C)** with addition of extract from SI + HEX, and **(D)** with addition of extract from SI + F. **(E)** Average NIH 3T3 fibroblast cell area (μm^2^) measurements over time (3 h period) for each tested extract. Data are presented as mean ± standard deviation. **(F)** Circularity index measurements over time (3 h period) for each tested extract. Data are presented as mean ± standard deviation. Scale bar = 100 μm. A separate linear regression model was run for each variable. (*) denote statistically significant differences between groups (p < 0.05)

The morphological changes and circularity index of NIH 3T3 fibroblasts under the experimental conditions are presented in Fig. 9. As shown in Fig. 9A, fibroblasts displayed typical elongated shape under control conditions. Cells exposed to SI (Fig. 9B), SI + HEX (Fig. 9C), and SI + F (Fig. 9D) extracts exhibited similar morphology compared to the control. Figure 9E shows changes in cell area over 3 h, all groups demonstrated an increase in cell area over time. This indicates that fibroblasts were able to attach and spread on the culture surface. The control and SI groups showed comparable increases in cell area, whereas the SI + F and SI + HEX groups exhibited slightly slower area expansion.

Panel 9 F shows changes in the circularity index over time (3 h) for each group. The circularity index is a measure of cell shape, where a value of 1 indicates a perfect circle, and values less than 1 indicates a more elongated shape. All groups exhibit a decrease in the circularity index over time, indicating a change in cell shape towards a more elongated morphology. The control and SI groups showed a similar decrease, while SI + F and SI + HEX exhibited a slightly slower reduction in circularity. Overall, these differences were not statistically significant. All tested extracts (SI, SI + F, and SI + HEX) did not impair fibroblast attachment or spreading on culture dishes, consistent with previously reported observations (Werner et al. 2007; Asare et al. 2013; Addis et al. 2020; Sumaiya et al. 2022; Morrow and Mutch 2023). This observation was further confirmed by a qualitative cytotoxicity test: MEM elution study with the L929 cell line (Table S1).

### Hemolysis and cytokine expression

To assess the biocompatibility of the material, hemolysis and cytokine expression was analyzed using whole human blood exposed to SI, SI + HEX and SI + F.

Expression levels of inflammation-associated cytokines in serum collected after 24 h of exposure to the tested voice prosthesis fragments (Panel B); SI (Provox), SI + HEX, and SI + F. A separate linear regression model was applied to each cytokine; ns-not significant.

**Fig. 10:**
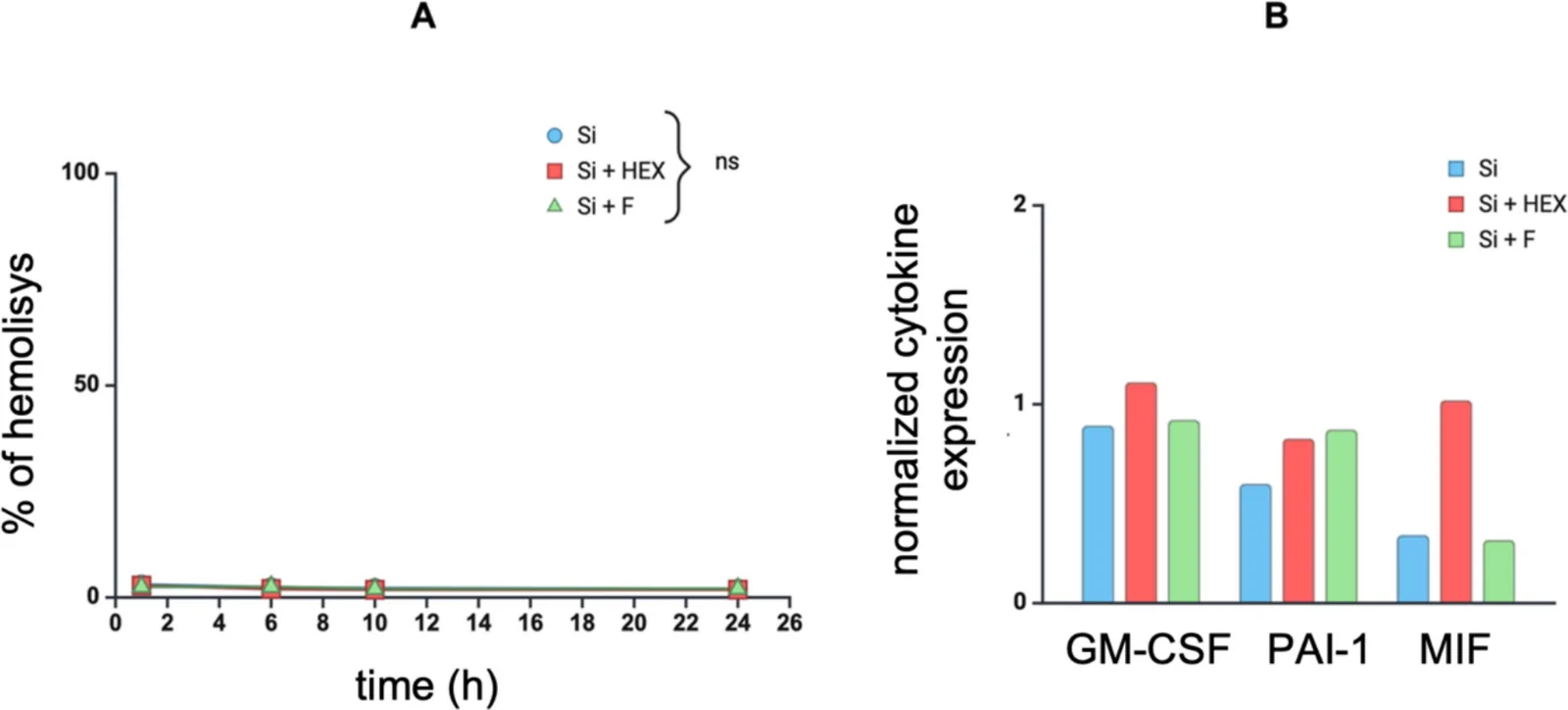
Hemolysis and cytokine expression following exposure to SI, SI + HEX, and SI + F. Percentage of red blood cell hemolysis in human whole blood after incubation with fragments of the tested voice prosthesis materials (Panel A); SI (Provox), SI + HEX, and SI + F—for 1, 6, 10, and 24 h. Data are presented as mean ± SD from three independent replicates

Panel A of Fig. 10 illustrates the percentage of hemolysis in whole blood samples incubated with VP fragments (SI, SI + HEX, SI + F) over time (1 h, 6 h, 10 h, 24 h). All three materials show a low capacity to induce hemolysis, with values remaining below 4%, throughout the incubation period. Panel B presents the normalized expression of granulocyte–macrophage colony-stimulating factor (GM-CSF), plasminogen activator inhibitor 1 (PAI-1), and macrophage migration inhibitory factor (MIF) in serum after 24 h of incubation with VP fragments. Among the tested materials, SI fragments exhibit the highest ability to induce expression of GM-CSF, compared to SI + HEX and SI + F which produced lower responses. All three materials elicited comparable levels of PAI-1 expression. For MIF, SI + HEX induced a very slightly higher response than the other materials. Overall, all materials displayed minimal hemolytic activity, indicating good biocompatibility. However, the differences in cytokine responses, may indicate varying potential for inducing an inflammatory/immunomodulatory response (Xu et al. 2019; Vuttaradhi et al. 2022).

### Durability of tested materials

The study revealed that SI exhibited the highest tensile strength among the tested materials, outperforming SI + HEX and SI +. The tensile strength for SI reached 9.8 MPa, this represents an increase of 0.59% compared with SI + HEX and 2.67% compared with SI + F. As part of the mechanical tests, Shore A hardness was also measured. SI showcased hardness values approximately 0.64% and 1.73% higher than those of SI + HEX and SI + F, respectively. These findings are consistent with the tensile strength results, with SI demonstrating the greatness hardness and SI + F exhibiting the lowest.

**Figure S3** presents mechanical parameters: tensile strenght (MPa), percentage of elongation at breakpoint and Shore A hardnesses scale of different type of prosthesis material (SI, SI + HEX and SI + F).

### Contact angle and surface free energy (SFE)

The contact angle analysis indicates that all tested silicone surfaces exhibit near-hydrophobic behavior, with values slightly above 80°. The applied surface modifications did not substantially alter material wettability. In general, hydrophobic surfaces (contact angles > 90°) tend to promote microbial adhesion due to favorable interactions between hydrophobic microbial cell walls and hydrophobic substrata, which reduce repulsive interfacial forces. However, the SFE calculations show that surfaces modified with fluorine and QAS exhibit higher SFE compared with unmodified SI. This increase in SFE suggests that the modified surfaces may be less susceptible to microbial colonization, as microorganisms with hydrophobic cell walls are less likely to adhere to surfaces with higher surface energy (Muhammad et al. 2020). **Figure S4** presents the contact angle measurements and surface free energy (SFE) values for the different prosthesis materials (SI, SI + HEX, and SI + F).

### Thermogravimetry (TGA)

To evaluate the activation energy associated with material degradation, TGA analyses were performed for SI, SI + HEX, and SI + F both alone and following biofilm cultivation. From the TGA curves, the temperatures corresponding to 1% and 5% mass loss were identified. Measurements were conducted at three heating rates (5, 10, and 20 °C/min) to enable calculation of the activation energy of thermal decomposition (Ea). The thermal decomposition analysis showed that the temperatures corresponding to 1% and 5% mass loss were higher in samples examined after biofilm cultivation for all tested materials (**Figure S5 panels A–C**). In contrast, the calculated activation energies were lower for biofilm-exposed samples compared with their respective controls. The most pronounced differences were observed for the following materials: a) SI: 324.2 kJ/mol (SI) and 215.7 kJ/mol (SI + biofilm), b) SI + HEX: 310.4 kJ/mol (SI + HEX) and 276.3 kJ/mol (SI + HEX + biofilm), c) SI + F: 298.4 kJ/mol (SI + F) and 298.2 kJ/mol (SI + F + biofilm). Overall, biofilm cultivation resulted in a reduction of activation energy, indicating that less energy is required to initiate thermal decomposition of the materials after microbial exposure (**Figure S5 panel D**).

## Discussion

A growing number of publications confirm the continued use of silicone in the development of novel therapeutic and diagnostic methods, including electrochemical biosensors for glucose monitoring, neuronal activity recording, and detection of tumor biomarkers and small molecules (Muhammad, Song et al. 2025). Silicon remains a widely used material due to its biocompatibility properties and high resistance to environmental factors (Moretto, Schulze et al.). However, despite these advantages, silicone implantable devices are susceptible to microbial colonization and biofilm formation, which significantly limits their clinical performance. Microbial colonization of indwelling medical devices represents a serious clinical concern, as mixed bacterial–fungal biofilms can lead to device failure, shorten implant lifespan, and contribute to complications such as aspiration pneumonia or bloodstream infections (Nobbs et al. 2010; Peters et al. 2012; Talpaert et al. 2015; Spałek et al. 2020).

An important aspect of understanding biofilm-associated complications is identifying the microorganisms involved and elucidating their mechanisms of invasion. Bacteria contribute to biofilm establishment and persistence through multiple immune-evasion strategies, including the production of polysaccharide capsules or surface proteins that inhibit phagocytosis or neutralize chemotactic signals, thereby impairing macrophage recruitment to the infection site. Once embedded within the biofilm matrix, bacterial cells can further adapt by altering their metabolic activity and gene expression profiles, enhancing their resistance to macrophage-mediated clearance.

Fungal pathogens also play an important role in biofilm formation on prosthetic materials. These organisms are capable of adhering to and penetrating device surfaces, aided by hydrolytic enzymes that degrade silicone and other material components. This enzymatic activity not only facilitates fungal invasion but also enables access to nutrients, supporting the development of deep, structured biofilms that are particularly difficult to eradicate.

Consistent with these mechanisms, previous studies have demonstrated that biofilms on VPs commonly comprise both bacterial and fungal cells (Somogyi-Ganss et al. 2017; Galli et al. 2018). In one investigation involving 42 explanted VPs, microbiological analysis revealed that only three devices harbored exclusively fungal colonization and five showed solely bacterial colonization, while the majority exhibited mixed microbial communities (Somogyi-Ganss et al. 2017). These findings underscore the polymicrobial nature of VP-associated biofilms and highlight the need for therapeutic strategies targeting both bacterial and fungal components.

Biocompatibility is another critical factor in selecting appropriate materials for VPs. Silicone modifications can influence its interaction with surrounding tissues and may interfere with local regenerative processes. Commonly evaluated parameters, such as fibroblast proliferation, cytokine expression, and hemolytic potential, provide insight into the material’s compatibility with biological systems. Fibroblasts are central to wound healing, and their ability to proliferate and migrate is essential for effective tissue repair. Elevated expression of pro-inflammatory cytokines, including IL-1, IL-6, IL-8, and TNF-α, may signal an adverse inflammatory response to the implanted material (Yang et al. 2023). Moreover, implantation of medical devices can induce hemolysis and contribute to blood flow disturbances (Avci et al. 2021), undermining the importance of minimizing such effects through careful material engineering.

In this context, silicone-based materials must be designed to support fibroblast activity while avoiding hemolytic effects, while also maintaining mechanical properties which are equally important; an ideal VP material should exhibit sufficient flexibility and durability to withstand continuous mechanical stress during phonation and swallowing. Notably, studies have shown that silicone membranes, particularly those incorporating surface modifications, demonstrate hemocompatibility profiles comparable to Teflon and stainless steel, characterized by low coagulation and complement activation as well as reduced platelet adhesion. These features have positioned surface-modified silicone as a promising candidate for applications such as miniaturized renal replacement systems (Muthusubramaniam et al. 2011), and they may be similarly advantageous for VP design.

The development of such optimized materials remains challenging but essential, as improvements in biocompatibility and mechanical performance have direct implications for the lifespan of VPs and the overall quality of life of patients who rely on them.

Our findings show that modified silicone surfaces (SI + HEX and SI + F) can effectively reduce biofilm formation. This reduction likely arises from multiple mechanisms involving alterations to the material’s surface properties and its interactions with microorganisms. This effect is likely driven by changes in key surface properties, particularly hydrophobicity and surface free energy, which play central roles in microbial adhesion. Previous work has demonstrated that altering these parameters can significantly influence silicone’s susceptibility to colonization (Litzler et al. 2007, Das et al. 2022, Krzywicka, Szymańska et al. 2022, Oda and Tanikawa 2022). Understanding these mechanisms is essential for refining silicone modifications to better prevent biofilm formation on VPs.

The relevance of such strategies is further supported by studies using quaternary ammonium salts (QAS) on other medical materials. For example, covalent attachment of QAS to titanium alloys used in orthopedic and dental devices has produced surfaces with strong anti-adhesion activity against *E. coli* and *S. aureus.* When combined with oleic acid, these modifications substantially alter surface characteristics and yield promising antimicrobial behavior (Celesti et al. 2022). Together, these findings highlight the potential of targeted chemical functionalization to enhance the antimicrobial performance of silicone and other biomaterials used in indwelling devices.

Ongoing research continues to focus on enhancing silicone’s resistance to biofilm formation. One of these being coating VPs with chlorhexidine-releasing varnish which has been shown to effectively inhibit biofilm development (Gross et al. 2022). Another study has introduced a microwave-assisted method for forming Si–S bonds on porous silicon which enables rapid, efficient surface functionalization while preserving porosity, expanding its potential for medical and drug-delivery applications (Oh et al. 2023). Additional work has shown that incorporating a double-substituted bornylsiloxane-based crosslinking agent can impart antimicrobial properties to silicone rubber (Chen et al. 2025). Furthermore, silicones containing 2% 1-dodecyl-3-methylimidazolium tetrafluoroborate or 5% polyhexamethylene guanidine dodecylbenzenesulfonate exhibit strong antimicrobial activity against common healthcare-associated pathogens, with minimal leaching (Morco et al. 2022). Collectively, these findings highlight the promise of chemical and structural silicone modifications for mitigating biofilm formation and reducing infection risks in indwelling silicone-based medical devices.

One of the earliest approaches to extending VP lifespan involved repeated argon-plasma treatment of silicone rubber. However, in vivo evaluation of the “Groningen Button” VP showed that biofilm formation was actually lower on the untreated prosthesis (Everaert, van de Belt-Gritter et al. 1998). The type of VP also influences biofilm susceptibility. In vitro studies demonstrate that the Provox ActiValve and Blom Singer Advantage prostheses exhibit significantly reduced biofilm formation compared with other devices and with unmodified silicone. The incorporation of materials such as silver oxide and Teflon in valve components may additionally contribute to long-term biofilm reduction (Leonhard et al. 2018).

This study has several limitations. Future work should investigate biofilm formation by additional oral microorganisms to more comprehensively assess the effects of the silicone modifications. Evaluating biofilm development over longer periods (beyond 14 days) is necessary to determine long-term performance. Finally, correlating these in vitro findings with clinical outcomes in a larger, prospective trial will be essential to validate the clinical relevance of these modifications.

## Conclusion

Our research confirms that currently used VPs undergo substantial microbial colonization, leading to considerable biofilm formation, although the extent varies among individuals. Quaternary ammonium salt–functionalized and fluorinated silicone modifications significantly reduce biofilm development, suggesting potential improvements in VP lifespan, complication rates, and patient outcomes. However, conducting experiments using biofilms formed solely by *Candida* spp. is a methodological limitation of our study and does not reflect the complexity of mixed biofilms on patients' VPs in vivo, which should be taken into account when interpreting the results.

While none of the tested materials impaired fibroblast adhesion, both modifications subtly affected cell spreading and morphology, which could influence tissue healing. Additionally, these surface treatments altered material durability, increasing surface free energy and reducing activation energy in the presence of biofilm.

Overall, these findings indicate that targeted surface modifications can effectively limit biofilm formation on VPs. However, further work is needed to refine these approaches to ensure optimal antimicrobial performance without compromising mechanical properties or biocompatibility. Continued research will be essential for developing durable, safe, and clinically effective strategies to mitigate biofilm-related device failure.

## Supplementary Information

Below is the link to the electronic supplementary material.

Supplementary file1 (DOCX 2.43 MB)

## Data Availability

The original contributions presented in the study are included in the article, further inquiries can be directed to the corresponding author.
