# Supplementary material for: Inhibiting biofilm growth on ammonium salt-functionalized or fluorinated voice prostheses silicone
Source: Appl Microbiol Biotechnol. 2026 Jun 30;110:279. doi: 10.1007/s00253-026-13904-z (PMC13586127; doi:10.1007/s00253-026-13904-z)
Supplement: Supplementary file 1 [file 253_2026_13904_MOESM1_ESM.docx]

**Supplementary material**

**Inhibiting biofilm growth on ammonium salt-functionalized or fluorinated voice prostheses silicone**

**Applied Microbiology and Biotechnology**

Sławomir Okła^1,2,^ *, Szczepan Kaliniak^1^, Jakub Spałek^1, 2^, Katarzyna Piotrowska^3^, Dawid Łysik^4^, Piotr Deptuła^5^, Konrad Żochowski^6^, Michał Dutkiewicz^7^, Joanna Mystkowska^4^, Urszula Wnorowska^6^, Robert Bucki^2,5,6^, Bonita Durnaś^1,2^, Monika Madej^3^, Hieronim Maciejewski^7,8^, Stanisław Góźdź^1,2^

^1^ Holy Cross Cancer Center, Artwińskiego 3, 25-734 Kielce, Poland

^2^ Institute of Medical Science, Collegium Medicum, Jan Kochanowski University of Kielce, IX Wieków Kielce 19A, 25-317 Kielce, Poland

^3^ Faculty of Mechatronics and Mechanical Engineering, Kielce University of Technology, Tysiąclecia Państwa Polskiego 7, 25-314 Kielce, Poland

^4^ Institute of Biomedical Engineering, Bialystok University of Technology, Białystok, Poland

^5^ Independent Laboratory of Nanomedicine, Medical University of Białystok, 15-222 Białystok, Poland

^6^ Department of Medical Microbiology and Nanobiomedical Engineering, Medical University of Białystok, 2C A. Mickiewicz Str., 15-222 Białystok, Poland

^7^ Poznań Science and Technology Park, Adam Mickiewicz University Foundation, Rubież 46, 61-612 Poznań, Poland

^8^ Faculty of Chemistry, Adam Mickiewicz University, Uniwersytetu Poznańskiego 8, 61-614 Poznań, Poland

*Corresponding author: Sławomir Okła; slawomir.okla@onkol.kielce.pl

**Figure S1**

*Figure S1. Cell viability of Candida albicans colonizing polymeric voice prostheses (SI, SI+HEX, and SI+F; 72 h incubation) assessed by live/dead staining. Fluorescence microscopy was used for image acquisition. Live cells are indicated in green (SYTO 9) in panels B, E, and H, whereas dead cells are shown in red (propidium iodide) in panels C, F, and I. Panels A, D, and G show optical images of prosthesis surfaces. The mean number of cells attached to prosthesis fragments, together with the standard deviation, is presented in panels B, E, and H.*

In this study, voice prosthesis fragments (SI, SI+HEX, and SI+F) were incubated with a C. albicans suspension prepared in medium with an optical density of 0.5 OD600. The samples were incubated for 72 hours in Sabouraud broth (Sigma-Aldrich, Germany) at 37°C. After incubation, the prosthesis fragments were gently washed with PBS to eliminate non-adherent cells. The samples were then immersed in PBS and stained with the Live/Dead viability kit (SYTO 9/propidium iodide) according to the manufacturer's protocol (Thermo Fisher Scientific, USA). Staining was performed for 15 minutes in the dark at room temperature. After staining, the samples were immediately analyzed under a fluorescence microscope. Live cells on the prostheses were visualized by green fluorescence (SYTO 9), while dead cells or cells with damaged cell membranes showed red fluorescence (propidium iodide PI). The results of this study confirm the significant limitation of the growth of the Candida biofilm on the surface of silicone functionalized with both ammonium salts and fluorine (reduction of fluorescence intensity - green color) as well as the lack of staining of potentially necrotic microorganism cells, which suggests their detachment or a much stronger inhibition of growth/adhesion than a direct fungicidal effect.

**Figure S2**

*Figure S2. Quantitative analysis of C. albicans using a calibrated loop. Representative plates with linear streak cultures, including mean cell counts (CFU/cm³) and standard deviations. Panel A -Si; panel B - SI + HEX; panel C - SI + F. A 98.0% and 99.95% reduction in the number of C. albicans-colonizing cells on the surfaces was observed for SI + HEX and SI + F, respectively, compared to the SI control samples*.

We quantified the growth of C. albicans colonies using a line culture method. After 72 hours of biofilm growth, the voice prosthesis fragments were sonicated in PBS after incubation, and two serial dilutions were made. Line culture was then performed using a calibrated 0.01 ml loop. Colonies were counted after 24 hours of incubation. The percentage reduction in the number of cells colonizing the prosthesis surface was determined.

**Figure S3**

*Figure S3****.*** *Mechanical parameters of tested voice prosthesis. Tensile strength [MPa] (Panel* ***A****), percentage of elongation at break (Panel* ***B****) and Shore A hardness (Panel* ***C****). The statistical analysis was evaluated by Tukey's multiple comparison test. (*) denote statistically significant differences between groups (p<0.05), ns - no significant difference.*

**Figure S4**

*Figure S4. Contact angle and surface free energy. Panel* ***A****: results of contact angle measurements using water on the various surfaces. Panel* ***B:*** *calculated surface free energy of the surfaces based on the Zisman model. The statistical analysis was evaluated by Tukey's multiple comparison test. (*) denote statistically significant differences between groups (p<0.05).*

**Figure S5**

**A**

SI	SI + biofilm

**B**

SI + HEX	SI + HEX + biofilm

**C**

SI + F	SI + F + biofilm

**D**

***Figure S5.*** *TGA graphs of example silicone/modified silicone (left graphs) compared to silicone/modified silicone after biofilm removal (right graphs) from representative prostheses (panels A-C). Panel (D) shows silicones activation energy, * p ≤ 0.05.*

# **Table S1**

# *Table S1. Cytotoxicity test results of silicone functionalized with fluorine (SI+F) and ammonium salts (SI+HEX).*

| **Sample name/parameter** | **SI+F** | **SI+ HEX** |
| --- | --- | --- |
| **Cell line** | L929 | L929 |
| **Cell passage** | 32 | 32 |
| **Extraction / culture medium** | Supplemented MEM | Supplemented MEM |
| **Extraction conditions** | 24±2 h; 37±1°C | 24±2 h; 37±1°C |
| **Extraction ratio** | 3 cm²/mL | 3 cm²/mL |
| **Negative control** | HDPE | HDPE |
| **Positive control** | Latex | Latex |
| **Blank** | MEM + 10% FBS + 4 mM Glutamax + penicillin/streptomycin | MEM + 10% FBS + 4 mM Glutamax + penicillin/streptomycin |
| **Cytotoxicity criterion** | grade ≥ 3 | grade ≥ 3 |
| **Blank result** | 0 (valid) | 0 (valid) |
| **Negative control result** | 0 (valid) | 0 (valid) |
| **Positive control result** | 4 (valid) | 4 (valid) |
| **Test sample result** | 0 | 0 |
| **Classification** | Non-cytotoxic | Non-cytotoxic |

We performed standardized cytotoxicity tests in accordance with the requirements of ISO 10993-5:2009(E) and ISO 10993-12:2021(E) using the qualitative cytotoxicity test: MEM elution assay. The test materials were SI+F and SI+HEX samples. Both samples were stored at 18–30°C. The L929 cell line, cultured at 37±1°C and an atmosphere of 5±0.1% CO₂, was used as the test system. Cells were maintained in MEM supplemented with 10% FBS, 4 mM GlutaMAX, 100 IU/mL penicillin, and 100 µg/mL streptomycin. Before the experiment, samples were disinfected with alcohol, dried for 30 minutes in a laminar flow cabinet, and then cut into smaller fragments. Extracts were prepared in supplemented MEM according to ISO 10993-12. For both materials, a surface-to-volume ratio of 3 cm²/mL was used, extraction time was 24±2 h, and temperature was 37±1°C. Controls were prepared in parallel: negative HDPE, positive latex, and a blank consisting of culture medium only. The extracts were not filtered or centrifuged and were used immediately after preparation. Triple replicates of L929 cell monolayers were used for the study, incubated with 600 µL of the respective extracts. After incubation, 100 µL of a staining solution prepared by mixing Trypan Blue with supplemented MEM in a 1:1 ratio was added to each well. Cytotoxicity was assessed microscopically based on a qualitative morphological scale according to ISO 10993-5. A score of ≥ 3 was considered a cytotoxic effect. The test acceptance criteria were met for both tested materials. The blank control and negative control achieved a grade of 0, while the positive control achieved a grade of 4, confirming the proper operation of the test system. The SI+F sample extract achieved a grade of 0, indicating no observable cytotoxicity in the qualitative test. Similarly, the SI+HEX sample extract also achieved a grade of 0. In both cases, the materials were classified as non-cytotoxic. Comparison of the results indicates that neither fluorination of the silicone nor the addition of ammonium salts in the tested material variant revealed any cytotoxic effect of the extracts on L929 cells under the test conditions (Please see table S1).

The obtained results indicate that both silicone materials analyzed exhibit a favorable biological profile in terms of preliminary in vitro cytotoxicity assessment. The lack of cytotoxicity for the SI+F sample suggests that the fluorine modification did not lead to the release of compounds capable of inducing morphological changes in L929 cells under elution test conditions. Similarly, the result obtained for the SI+HEX sample indicates that the presence of ammonium salts in this formulation did not result in detectable cytotoxicity of the extract. The validity of these observations is also influenced by the accuracy of the internal controls: the lack of reaction for the blank and HDPE and the strong response for the latex confirm the appropriate sensitivity of the method. However, it should be emphasized that the presented study was qualitative in nature and involved only the evaluation of material extracts in a single cell model. These results do not constitute full biocompatibility of the materials but constitute an important screening step. Further characterization should include, at a minimum, quantitative tests of cell viability/metabolism, analysis of cell adhesion and proliferation, and, depending on the intended application, further tests according to the ISO 10993 series.

**Table S2**

**Table S2.** Strength test parameters.

| **Parameter** | **Value** |
| --- | --- |
| Length of sample section | 50 mm |
| Sample thickness | 2 mm |
| Width of samples | 4 mm |
| Stretch speed | 5 mm/min |
